# Molecular Endoscopy for the Diagnosis and Therapeutic Monitoring of Colorectal Cancer

**DOI:** 10.3389/fonc.2022.835256

**Published:** 2022-02-25

**Authors:** Maximilian J. Waldner, Markus F. Neurath

**Affiliations:** ^1^ Department of Medicine 1, Friedrich-Alexander-Universität Erlangen-Nürnberg, Erlangen, Germany; ^2^ Deutsches Zentrum Immuntherapie (DZI), Friedrich-Alexander-Universität Erlangen-Nürnberg, Erlangen, Germany

**Keywords:** colorectal cancer, adenoma, endoscopy, molecular imaging, confocal endomicroscopy, Raman, fluorescence, near-infrared imaging

## Abstract

Colorectal cancer (CRC) is one of the leading causes of cancer related death in the western world. Its successful treatment requires early detection and removal of precursor lesions as well as individualized treatment of advanced disease. During recent years, molecular imaging techniques have shown promising results to improve current clinical practice. For instance, molecular endoscopy resulted in higher detection rates of precursors in comparison to conventional endoscopy in preclinical and clinical studies. Molecular confocal endomicroscopy allowed a further classification of suspect lesions as well as the prediction and monitoring of the therapeutic response. In this review, we summarize recent achievements for molecular imaging of CRC in preclinical studies, initial clinical trials and the remaining challenges for future translation into clinical practice.

## Introduction

Colorectal cancer (CRC) is among the leading causes of cancer-related mortality in the Western world. For instance, CRC has been estimated to result in more than 50.000 deaths in both men and women in the United States in 2021, making it the 3rd most common cause of tumor-related death in both genders (1). It is well accepted that most CRCs slowly develop from precursor lesions such as adenomatous polyps, which develop within the adenoma-carcinoma sequence, or polyps of the serrated pathway (2). Thus, endoscopic screening and removal of precursor lesions have been implemented into clinical routine for secondary CRC prevention and are recommended for patients over the age of 50 in the guidelines of most international and national professional societies (3). However, endoscopic screening for CRC is associated with relevant miss-rates for CRC precursors up to nearly 30% (4, 5). Therefore, there is an urgent demand for techniques improving the detection rate of CRC precursors within screening endoscopy.

A promising approach to improve the sensitivity for the detection of CRC and its precursors are molecular imaging techniques. Molecular imaging can be described as the non-invasive visualization of biochemical events at the cellular or even molecular level in cells, tissues or whole organisms (6). This can be accomplished with specialized imaging devices with or without targeted contrast agents. Depending on the molecular target that is visualized, molecular imaging techniques can provide temporospatial information on biochemical processes including cellular metabolism or the activation of certain pathways that are specific for a disease. If performed *in vivo*, molecular imaging approaches can provide important information in realtime and therefore improve decision making during diagnostic procedures. In the field of cancer diagnostics and therapy, molecular imaging has become an important research topic in recent years (7). This also applies to CRC, which has been used as a cancer role model for various molecular imaging approaches in the past. Although various imaging techniques including PET, SPECT, MRI and others have been applied, optical endoscopic imaging is the most used technique for the molecular evaluation of CRC. This can be attributed to the fact that molecular endoscopy can aid the endoscopic detection of precursor lesions and therefore improve diagnostic accuracy or can potentially help in the classification of suspect lesions, which then can directly be endoscopically removed within the same procedure. Therefore, this review will summarize the preclinical development and current clinical translations of molecular imaging endoscopy for the diagnosis and therapy of CRC.

## General Principles of Molecular Imaging

Due to the advances of biomedical research during recent decades, molecular mechanisms involved in the development of various forms of cancer including CRC could be understood more and more precisely. This knowledge not only enabled the development of new targeted therapeutics, but also showed that distinct molecular changes already occur at early stages of disease development. Consequently, it was proposed that the visualization of molecular alterations with dedicated imaging techniques could increase the sensitivity and specificity of cancer detection (6). An early example for such an approach was the detection of somatostatin receptors on neuroendocrine tumors with scintigraphy of radiolabelled octreotid about 20 years ago (8). Since then, molecular imaging approaches have been implemented for various techniques including tomographic imaging, ultrasound or optical imaging techniques such as endoscopy (9–11). Despite the fundamental differences of these imaging modalities, several principles of molecular imaging can always be applied. In general, molecular imaging visualizes the differential expression of disease-specific molecules. In most instances, exogenous labeling is required, as endogenous molecules cannot be detected specifically and with sufficient contrast. Exogenous labeling is usually performed with contrast agents that contain a ligand specific for the target of interest, which is linked to a reporter molecule (12). As optical molecular imaging is usually achieved through fluorescence detection, fluorochromes are used as reporter molecules. For preclinical molecular imaging, various fluorochromes with excitation/emission peaks in the visible or NIR spectrum are available. In contrast, only a limited number of fluorochromes such as fluorescein, indocyanine green (ICG) or IRDye 800CW have so far been used in clinical trials. Ligands include antibodies, peptides, enzymes, affibodies or lectins (13). Overall, choosing the right imaging technique, the optimal fluorochrome, ligand and molecular target for each clinical indication will be essential for successful molecular imaging. In the next chapters, successful strategies for CRC will be discussed.

## Molecular Targets of Colorectal Cancer

Whereas imaging techniques and contrast agents have been continuously improved during recent years, choosing the right target for molecular imaging approaches is still a challenge and the bottleneck for many applications. Several general characteristics of potential targets for molecular imaging techniques have been proposed (14). These include a high expression of the molecular target on cancer cells in comparison to the surrounding healthy tissue. Furthermore, molecules on the cell surface are preferred due to improved binding of the imaging probe. An important issue with optical imaging techniques is the low penetration depth. Therefore, imaging targets should be close to the imaging device, e.g. the luminal side is preferred for endoscopic applications. Another important aspect is the route of administration. Whereas luminal and cell-surface antigens are accessible by topical application of the imaging probe, other antigens require systemic (e.g. intravenous) administration of the probe, what could be associated with an increased risk for side effects (e.g. allergic reactions).

Initial studies on the molecular imaging of cancer focused on genes that were known to be dysregulated during tumor development. In this regard, CRC has lent itself to initial studies on molecular imaging, as it was one of the first tumors in which research succeeded in linking molecular pathways with cancer development. In the 1980s, researchers proposed that distinct genetic alterations are involved in the four steps of CRC development, which entail adenoma, carcinoma in situ, invasive and metastatic cancer (15). In 1990, Fearon and Vogelstein linked these stages of CRC development with mutations in specific molecules (16). These included mutations in the genes adenomatous polyposis coli (*APC*), Kirsten rat sarcoma virus (*KRAS*), and tumor protein 53 (*TP53*). Until today, these genes are considered as major tumor suppressor genes (*APC* and *TP53*) or oncogenes (*KRAS*) of CRC development, although mutations in other genes have subsequently been discovered within the adenoma-carcinoma sequence model. Because of these findings, initial studies aiming at the detection of CRC precursors with optical molecular imaging targeted molecular pathways that were associated with mutations occurring early in the adenoma-carcinoma sequence. As the genes mutated within the adenoma-carcinoma sequence are mostly intracellular and therefore not well suited for molecular endoscopy approaches, markers have been identified that are associated with a dysregulation of pathways of the adenoma-carcinoma sequence. For instance, Marten et al. developed an activatable probe for the proteinase cathepsin B for the detection of adenomas, as early occurring mutations in *APC* could induce the activation of proteolytic enzymes (17). They evaluated the probe in the widely used APCmin/+ mouse model of CRC and could show a specific activation of their cathepsin B probe in mouse adenomas using whole body fluorescence imaging. Subsequent studies used cathepsin B probes for endoscopic molecular imaging of adenomas in mice (18, 19). Another molecular target that has been used by several studies for the early detection of CRC is c-MET (tyrosine-protein kinase MET). c-MET is a tyrosine-protein kinase that is activated by growth factors such as hepatocyte growth factor/scatter factor within embryonic development, wound healing etc. (20). Aberrant (over-)expression of c-MET in CRC has already been recognized in early studies including the land-mark study by Fearon and Vogelstein (16). Due to its location at the cell membrane of epithelial cells, it is well accessible for molecular imaging probes. Other examples of early targets for molecular imaging approaches in CRC include carcinoembyonic antigen (CEA), cyclooxygenase 2 (PTGS2), Thomsen-Friedenreich antigen and others (21–25).

Despite these achievements with already established molecular targets of CRC, there has been a continuously increasing effort to identify new cancer biomarkers. In this regard, the use of phage display libraries to identify peptides binding to cancer cells has been a promising strategy. For instance, Hsiung et al. identified a septapeptide with specific binding to dysplastic colonic epithelial cells through a M13 phage library (26). The peptide was conjugated with the widely used fluorochrome fluorescein to perform molecular endoscopy of CRC precursors in mice *in vivo*. Similarly, De Palma et al. identified a heptapeptide that allowed the detection of dysplastic epithelial cells in inflammatory bowel disease (27). Another strategy is based on the bioinformatical evaluation of mRNA expression data of CRC samples. By evaluating microarray data from more than 600 samples with adenocarcinoma, adenoma and various controls for differentially expressed genes of cell surface proteins, Sewda et al. found six previously unrecognized markers of CRC that were further validated ex vivo in cell culture and on human tissue samples of CRC (14).

In contrast to polyps arising within due to the adenoma-carcinoma sequence, the development of serrated polyps is usually associated with mutations in the MAPK pathway or the CpG island methylator phenotype (CIMP). Therefore, endoscopic molecular imaging of serrated polyps might need other targets in comparison to polyps of the adenoma-carcinoma sequence. For instance, Joshi et al. developed a peptide that showed specific binding to human colorectal cancer cells with the V600E mutation of *BRAF*, which can frequently be detected in serrated polyps (28). If these markers can be used for molecular imaging of CRC, will need to be evaluated in further studies.

## Devices for Endoscopic Molecular Imaging

**Figure 1:**
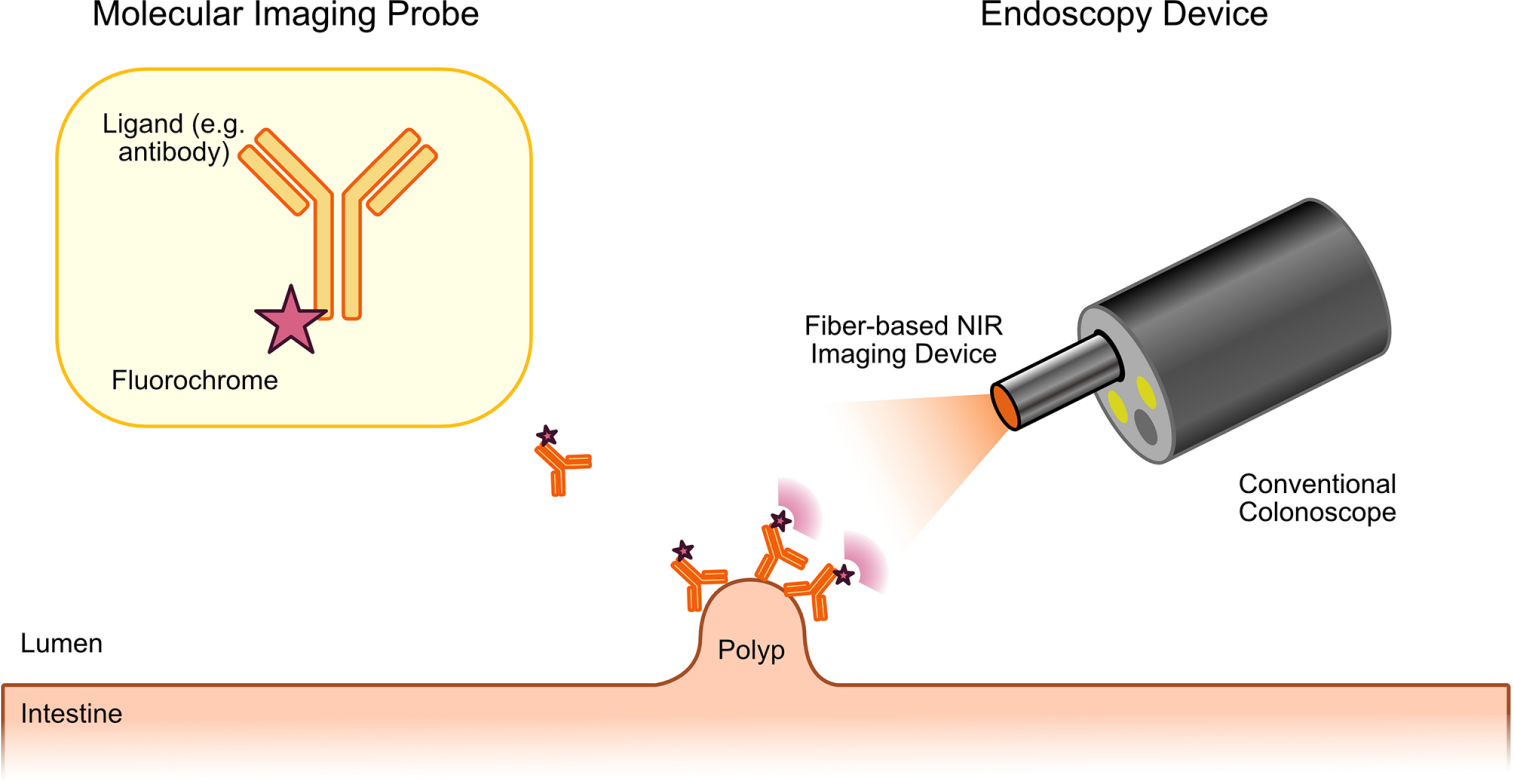
Principle of molecular endoscopy. A fluorescent probe (ligand molecule coupled to fluorochrome) is applied either topically or systemically during or before the endoscopic procedure. Fluorescence endoscopy is then performed with a dedicated fluorescence endoscope. This can either be a fiber-based device that will be fed through the working channel of a conventional endoscope or with fluorescence colonoscopes. The fluorescence endoscope will excite the tracer molecule, which has bound to the target molecule on the tumor precursor. Excited tracers will emit light with a longer wavelength that can specifically be detected by the fiber-based molecular endoscopy device enabling specific imaging of CRC precursors with high contrast.

As previously discussed, optical molecular imaging is mostly based on the detection of fluorescence. To enable endoscopic molecular imaging of CRC, this required the development of dedicated fluorescence endoscopes. In this regard, two main strategies were pursued. To improve the sensitivity for the detection of potential CRC precursors, widefield fluorescence endoscopes were required. In addition, confocal laser endomicroscopes (CLE) were developed to enable an *in vivo* microscopic evaluation of tissues including CRC. Both types of devices together could enable a two-step approach: widefield molecular endoscopy could be used as a red-flag technique for the detection of suspicious lesions, which could then further be characterized by microscopic molecular imaging (7). The first widefield fluorescence endoscopes were built to detect auto fluorescence. In autofluorescence imaging (AFI), endogenous fluorochromes in biological tissues are excited with light of the visible spectrum and the emitted light with longer wavelengths is detected by using specific filter sets (9, 13). Typical endogenous fluorochromes include flavin adenine dinucleotide (FAD), reduced nicotinamide (NADH), porphyrins or hemoglobin. Images generated by AFI endoscopes are pseudocolored representations of a mixture of all endogenous fluorochromes together. Although alterations of AFI signals can be detected in gastrointestinal cancers and their precursors (including CRC), the technique is limited due to low specificity and a high false-positive rate for cancer detection. However, its main advantage is the fact that no exogenous fluorochromes are required. In contrast, molecular imaging with exogenous probes offers the specific detection of single types of molecules with improved signal-to-noise ratio. Furthermore, it is not restricted to endogenous fluorochromes. In contrast to AFI endoscopes, where fluorescence is detected in the visible light spectrum, endoscopes for molecular imaging of exogenous fluorochromes were mostly developed to detect fluorescence in the near-infrared (NIR) light spectrum (12, 29). This approach offers two advantages. On the one hand, there are less endogenous molecules absorbing light in the NIR spectrum (optical window of biological tissues) resulting in a higher penetration depth. On the other hand, detection of fluorescence in the NIR spectrum enables the simultaneous imaging of the visible light providing a combination of white light and fluorescence endoscopy. First NIR endoscopes were described at the beginning of the 20th century. For instance, Funovics et al. described a fiber-based multichannel endoscope for NIR and white light endoscopy for the use in mice (30). This or similar systems have subsequently been used in various preclinical studies. In 2013, Garcia-Allende described two NIR devices that were based on commercial, clinical grade endoscopes (31). These included an integrated NIR fiber colonoscope as well as a semi-disposable NIR fiber system with small diameter that could be fed through a conventional video colonoscope (**Figure 1** illustrates such a technical setup). A first clinical study using NIR endoscopy for the detection of CRC precursors in humans was published in 2015 by Burggraaf et al. (32).

In contrast to widefield fluorescence endoscopes, CLE devices were built to deliver microscopic information about suspect lesions during endoscopy. Initially, two different systems have been developed and used in preclinical as well as clinical trials (12). In one system, the CLE device was integrated into the tip of a clinical videoendoscope (eCLE, Pentax, Tokyo, Japan, no longer for sale). In the other system, a probe-based CLE device (pCLE, Mauna Kea Technologies, Paris, France) with flexible mini-probes was developed, which can be fed through the working channel of standard endoscopes. Both CLE devices rely on the detection of fluorescence excited at 488 nm and therefore can visualize fluorochromes such as fluoroscein or acriflavine. An initial study using the eCLE system for the evaluation and classification of adenomas and CRC in humans has been performed by Kiesslich et al. in 2004 (33). However, CLE has not been used for targeted molecular imaging in this study, as intravenously fluorescein sodium was used as a contrast agent, which only provides structural and not molecular information. Later studies also used CLE devices for molecular imaging of CRC as discussed subsequently.

## Detection of CRC Precursors Lesions With Molecular Endoscopy

The first studies that evaluated the potential of molecular imaging to identify precursor lesions of CRC were mainly focused on showing an upregulation of the molecular target and the possibility to visualize this upregulation through the detection of fluorescence. For instance, in the previously mentioned study by Marten et al., it was shown that Cathepsin B, the target of the used activatable protease sensing NIR probe used in this study, is upregulated in adenomatous polyps of the APCMin/+ mouse model of CRC (17). Furthermore, it was found that the cathepsin reporter probe specifically binds to intestinal adenomas and fluorescent tumors of mice can be detected with sufficient background to noise ratio by busing a widefield fluorescence imaging system. Subsequent studies validated the specific binding and potential to identify tumors in mouse models of CRC with cathepsin B activatable probes using custom-made molecular endoscopy devices (18, 19). Tjalma and co-workers initially analyzed the specific binding of anti-vascular endothelial growth factor (VEGF) and anti-epidermal growth factor receptor (EGFR) antibodies to hyperplastic (benign) polyps, various types of adenomas and colorectal cancer samples (34). Both VEGF and EGF are growth factors that are involved in the pathogenesis of CRC. Whereas VEGF is regarded as the most important mediator of tumor angiogenesis through the stimulation vascular endothelial cell proliferation, migration, survival etc., EGF is important for the regulation of intestinal epithelial cell growth, e.g. during wound healing, but also tumor development. Interestingly, the study of Tjalma et al. showed superior binding of anti-VEGF antibodies to all stages of CRC development (34). Therefore, the same group performed a first clinical study using NIR-labeled anti-VEGF-antibodies for the endoscopic detection of CRC precursors in patients with familial adenomatous polyposis, a hereditary disease causing high adenoma and CRC incidence due to a mutation in the APC gene (35). In this study, specific binding of anti-VEGF antibodies to adenomas was shown, highlighting the potential of molecular endoscopy for the early detection of CRC precursors. Another study by Foersch et al, used CLE for the detection of VEGF expression in CRC mouse models and could also show the specific expression of VEGF in xenograft mouse models of CRC and humans tissue samples with molecular endomicroscopy (36). Similar studies have been performed for cyclooxygenase 2 (PTGS2), g-glutamyltranspeptidase, integrin avb3 and other molecules (22, 23, 37, 38). In contrast to these studies, which used widefield endoscopy, the previously discussed study by Hsiung et al. used a fluorescently labeled septapeptide for the detection of colonic dysplasia with CLE (26). The septapeptide showed 81% sensitivity and 78% specificity for dysplasia in comparison to surrounding normal mucosa. A similar approach was used by De Palma et al. for the detection of dysplasia in patients with ulcerative colitis, which have an increased risk for CRC development due to chronic mucosal inflammation (27). However, all these studies did not evaluate how molecular endoscopy approaches compare to standard white light endoscopy for the detection of CRC precursors.

In this regard, Schwegmann et al. used a NIR matrix metalloproteinase specific probe for the endoscopic detection of colon tumors induced through the administration of azoxymethane and dextrane sodium sulfate (AOM+DSS) in mice (39). They evaluated the number of tumors detected with conventional white light endoscopy in comparison to fluorescence stereoscopy. Of note, fluorescence stereoscopy allowed the detection of more tumors than white light endoscopy. A first study comparing molecular endoscopy to white light endoscopy in humans was published by Burggraaf et al. in 2015 (32). The used a 26-amino acid peptide with specific binding to c-MET labeled with a NIR cyanine dye. Using this NIR probe, molecular endoscopy was able to detect all neoplastic polyps that were detected with white light endoscopy and even nine additional polyps that had not been diagnosed with white light endoscopy.

## Evaluating the Response to Therapy

Besides the detection of CRC and its precursors, molecular endoscopy has also been proposed for the prediction or monitoring of the response to anti-cancer therapy. For instance, Manning et al. analyzed the use of two NIR-labeled probes targeting EGFR and annexin V (40). Anti-EGFR therapeutics such as the anti-EGFR antibody cetuximab belong to the standard therapy of advanced CRC. However, not all patients respond to these therapeutics. Therefore, evaluating the expression of EGFR with molecular endoscopy was proposed to enable the identification of patients that would benefit from anti-EGFR therapeutics. Furthermore, annexin V, a marker for apoptosis, was used to detect therapy induced cell death of cancer cells. The authors could show that the NIR-labeled EGFR probe could provide information about the EGFR expression in a CRC xenograft model *in vivo* (40). Following cetuximab administration, binding of the NIR-EGFR probe was reduced, suggesting specific binding of cetuximab. Furthermore, accumulation of the NIR-annexin V probe correlated with apoptosis of CRC cells in immunohistochemistry for caspase 3. Whereas this study did not use an endoscopic device for molecular imaging, but a wiedefield whole body imaging system, a study by Goetz et al. used CLE to evaluate the binding of a fluorescently labeled anti-EGFR antibody to human CRC cell lines in a xenograft mouse model and human CRC tissue samples (41). The data from molecular CLE were correlated with immunohistochemistry and could confirm the data by Manning et al. In a follow-up study, Goetz et al. directly labeled cetuximab with the fluorochrome Alexa Fluor 488 to compare the response to cetuximab treatment with molecular CLE in a xenograft CRC mouse model (42). In fact, the response to cetuximab could be predicted by anti-EGFR molecular imaging at the time of treatment initiation.

Whereas these studies evaluated the possibility of molecular endoscopy to predict the response to therapy, Miyamoto et al. used fluorescently labeled anti-EGFR antibodies to monitor the response to therapy after treatment initiation (43). In this study, nude mice bearing human colorectal cancer cells were treated with the standard chemotherapeutic 5-fluorouracil (5-FU) and subsequently evaluated with molecular endoscopy for EGFR expression. Interestingly, EGFR signal intensity was reduced in 5-FU-treated animals, reflecting the amount of living cancer cells. **Table 1** summarizes preclinical and clinical studies on the diagnosis and therapy of CRC using molecular endoscopy.

**Table 1:** Preclinical and clinical studies on molecular endoscopy in CRC.

Type of application	Technique	Molecular target	Type of trial	Ref.
**Identification of precursor lesions**	Ex vivo widefield NIR imaging	Cathepsin B	Preclinical – APCMin/+ mouse model	(17)
	Widefield NIR endoscopy	Cathepsin B	Preclinical – CT26 mouse model	(18)
	**Confocal endomicroscopy**	**Septapeptide**	**Clinical pilot study**	(26)
	Confocal endomicroscopy	VEGF	Preclinical – APCMin/+ mouse model, SW480/SW620 mouse model, human tissue samples	(36)
	**Widefield NIR endoscopy**	**c-MET**	**Clinical pilot study**	(32)
	Widefield NIR endoscopy	MMP-2/MMP-9	Preclinical – AOM+DSS mouse model	(39)
	Widefield NIR endoscopy	Cathepsin B	Preclinical – AOM+DSS mouse model	(19)
	Ex vivo widefield NIR endoscopy	VEGF, EGFR	Preclinical - Human tissue samples, HCT116 mouse model	(34)
	**Widefield NIR endoscopy**	**VEGF**	**Clinical pilot study**	(35)
**Evaluation of response to therapy**	Widefield NIR imaging	EGFR, Annexin V	Preclinical - Human tissue samples, HCT116 mouse model	(40)
	Confocal endomicroscopy	EGFR	Preclinical – SW480/SW620 mouse model, human tissue samples	(41, 42)
	Widefield NIR imaging	EGFR	Preclinical – Xenograft mouse models	(43)

## Limitations and Future Directions

Despite these encouraging results, several limitations still restrict the use of molecular endoscopic imaging for the clinical management of CRC. For instance, regulatory requirements for the clinical approval of imaging probes are comparable to therapeutic drugs. However, due to a reduced revenue, industrial long-term interest in the development of imaging probes is rather weak (44). Additionally, it is not clear, if imaging probes that interfere with potential therapeutic targets might impact the future response to therapy. For instance, labelled antibodies could induce the development of host antibodies, which might later neutralize a therapeutic drug (e.g. antibodies neutralizing cetuximab following imaging with an anti-EGFR-antibody). Furthermore, genetic heterogeneity of cancers including CRC results in only low sensitivity and sensitivity of most approaches targeting single molecules. In 2015, 4 molecular subtypes of colorectal cancer were described, all of which can be characterized by the expression of specific molecular pathways (45). These molecular subtypes not only show differences regarding the prognosis or response to therapy, but also might prevent the diagnosis of CRC precursors in all patients by targeting only one molecule during molecular imaging. As research is continuing, we can speculate on even more molecular subclasses of CRC, which will require new concepts of diagnosis and therapy.

To overcome this problem, various strategies have been developed to allow the simultaneous evaluation of several molecules during molecular endoscopy. For instance, Miller et al. developed a multispectral fluorescence endoscopy system that provided excitation at three different wavelengths at the same time (46). Using this system, the authors successfully showed the detection of two CRC specific peptides at the same time in the APCmin/- mouse model of CRC. Luthman et al. even provided a fluorescence endoscopy system that covers 25 spectral bands with wavelengths between 659 and 891 nm (47). Unfortunately, the number of simultaneously detectable fluorochromes is limited due to spectral overlap of available fluorochromes. Therefore, Garai et al. proposed a multiplexing endoscopy system that uses surface-enhanced Raman spectroscopy (SERS) (48, 49). Raman spectroscopy provides information about inelastic scattering of molecules. The spectral peaks detectable with Raman spectroscopy are characterized by high specificity and narrow bandwidth. This enables the detection of various labels at the same time. As endogenous molecules only provide weak Raman signals, specific Raman-active molecules, which are coupled to ligands for the desired target molecules, are used during SERS. In the study be Garai et al., nanoparticles were used that can incorporate a targeting molecule together with the Raman-active molecules (48). These SERS molecules were then visualized with a rotating sideview widefield Raman imaging system, which enabled systematic imaging of the intestinal surface. The authors used this system to identify six different SERS nanoparticles in a hollow lumen phantom. In ex vivo porcine studies, cocktails of various SERS nanoparticles were injected into the mucosa and the individual amount of individual particles within the cocktails was quantified. These studies provide exciting possibilities for multi-molecular imaging of CRC. For instance, multiplexing molecular endoscopy approaches might not only improve the detection rate of precursor lesions, but also provide an initial molecular subclassification of newly detected CRCs, which could aid subsequent personalized management of CRC. However, further studies are required to validate these concepts *in vivo*.

In addition to multiplexing various molecular targets at the same time, another concept is the combination of complementary imaging techniques for the evaluation of CRC and its precursors. As optical fluorescence imaging, widefield and microscopy, are limited due to a limited penetration depth, other techniques are required to provide information about the three dimensional architecture of suspect lesion. In this regard, fluorescence endoscopy has been combined with optical coherence tomography (OCT) in several studies (21, 50). As OCT provides structural information with higher penetration depth, it could give information e.g. on infiltrative growth of CRC precursors.

## Conclusion

Nearly 20 years ago, initial studies proposed molecular imaging approaches as new diagnostic modalities for the early detection of colorectal cancer. Since then, efforts in the development of molecular imaging probes, fluorescence endoscopes and their usage resulted in initial clinical trials that propose that molecular endoscopy might be superior for the detection of CRC precursors during screening colonoscopy. Data from preclinical studies further show a usage for the prediction of therapy or therapeutic monitoring. However, as new insights are gained on the genetic heterogeneity of CRC and efforts for personalized therapy are constantly progressing, there is still a huge demand on further research about the full potential of molecular endoscopy in the clinical management of CRC. Recently developed multispectral systems that allow the simultaneous detection of various targets at the same time pose important steps for future applications. However, the identification of appropriate molecular targets of CRC development and biomarkers for a response to therapy is still the bottleneck for molecular imaging approaches.

## Author Contributions

MW and MN performed literature research, planned and wrote the manuscript. All authors contributed to the article and approved the submitted version.

## Conflict of Interest

The authors declare that the research was conducted in the absence of any commercial or financial relationships that could be construed as a potential conflict of interest.

## Publisher’s Note

All claims expressed in this article are solely those of the authors and do not necessarily represent those of their affiliated organizations, or those of the publisher, the editors and the reviewers. Any product that may be evaluated in this article, or claim that may be made by its manufacturer, is not guaranteed or endorsed by the publisher.
